# The metabolic basis of cognitive insight in psychosis: A positron emission tomography study

**DOI:** 10.1371/journal.pone.0175803

**Published:** 2017-04-17

**Authors:** Elisabetta Caletti, Giorgio Marotta, Giuseppe Del Vecchio, Riccardo A. Paoli, Michela Cigliobianco, Cecilia Prunas, Elisa Zugno, Francesca Bottinelli, Paolo Brambilla, A. Carlo Altamura

**Affiliations:** 1 Department of Psychiatry, University of Milan, Fondazione IRCCS Ca' Granda Ospedale Maggiore Policlinico, Milan, Italy; 2 Section of Nuclear Medicine, Fondazione IRCCS Ca' Granda Ospedale Maggiore Policlinico, Milan, Italy; 3 IRCCS “E. Medea”, San Vito al Tagliamento (PN), Italy; 4 Department of Psychiatry and Behavioural Neurosciences, University of Texas at Houston, Houston, Texas, United States of America; University of Colorado Denver School of Medicine, UNITED STATES

## Abstract

The purpose of this study was to investigate the relationship between cognitive insight and cerebral metabolism in patients suffering from psychosis. The Beck Cognitive Insight Scale (BCIS) was administered to 63 patients with psychosis undergoing Positron Emission Tomography investigation. The sample was divided into two groups considering the BCIS score. Data were analyzed using Statistical Parametric Mapping. Results: patients with low insight, compared to those with high insight, showed decreased metabolism in the right fusiform gyrus, left precuneus, superior temporal gyrus and insula bilaterally, as well as increased metabolism in the left orbito-frontal gyrus (all p<0.005). Our results suggest that reduced posterior (occipito-temporo-insulo-parietal) and increased anterior (orbitofrontal) cerebral metabolism may sustain low cognitive insight in psychosis.

## 1. Introduction

In psychiatric lexicon the term insight was introduced in order to define the degree of awareness in psychosis [1]. Specifically, the lack of insight is an essential and complex concept for diagnosis and treatment of psychosis [2,3,4,5,6]. Insight has been described as the patient’s ability to assess the nature and severity of his disease [7] and a multidimensional construct that can be related exclusively to some aspects of the disease [8]. Poor insight in schizophrenia (SZ) has been associated with bad compliance [9,10] and worse long term consequences of psychopathology [5,11]. However, lower insight level can also be found during manic episodes in bipolar disorder (BD) [12,13,14,15].

Previous studies explored the association between insight and cognitive performance in SZ [16,17], since these patients demonstrate significant deficits in many important cognitive domains [18,19,20,21]. Indeed, it has been consistently shown that a negative correlation exists between high need of medical care and low cognitive functions in severe psychiatric illness [22]. Neuroimaging studies have shown frontal lobe dysfunction related to illness unawareness in psychosis [23,24,25]. Sapara and colleagues [26] observed smaller prefrontal gray matter volume, while Gerretsen and colleagues [27] found that a cerebral asymmetry correlated with insight [a lower volume of dorsolateral prefrontal cortex (DLPFC), angular gyrus (AG), parietal lobe and insular cortex in the right hemisphere].

Recently, Nair and colleagues [28] highlighted the complexity of the insight construct, revealing some associations between neurocognition, “clinical” insight and “cognitive” insight. The latter is the metacognitive ability to reflect on our and others mental states to predict appropriate behaviour [29,30]. It is also described as the ability to use mental states in problem solving and interpersonal strategies, in addition to abilities of inferring emotions from others facial expressions, behaviors and actions [31]. Many previous studies found insight to be associated with metacognition deficits [32,33,34,35,36]; particularly self-reflectivity, empathetic perspective-taking and mastery were shown to be connected to awareness of illness and social functioning [37,38]. Buchy and colleagues [39] defined cognitive insight as “…an individual’s ability to understand their own reasoning processes, beliefs and judgments” and showed that healthy subjects have better cognitive insight (higher R and lower C) compared to psychotic patients. This deficiency also refers to a limited ability in evaluating anomalous experiences and erroneous inferences [40] (e.g. impaired objectivity about cognitive distortions, loss of ability to put into perspective, difficulties in correcting feedback and overvaluation of conclusions).

The Beck Cognitive Insight Scale (BCIS) [41] has been developed to evaluate patients’ self-reflectiveness and overconfidence about their interpretations of experiences. Indeed, these authors showed that the BCIS R-C index (the difference between self-reflectiveness score “R” and self-certainty score “C") is correlated to the Unawareness of Mental Disorder Scale (SUMD) subscale “awareness of having a mental disorder”. Indeed subjects with better cognitive insight (higher R-C index) show a higher awareness of illness, but the two measures are not equivalent, because they represent two different dimensions of insight [42]. The BCIS R-C index could represent a satisfactory measure of cognitive insight in patients with psychosis, as several studies have previously demonstrated [41,43,44,45]. Interestingly, reduced self-reflectiveness and overconfidence of experiences interpretation may be a form of neuropsychological deficit [46] that, from a neurobiological point of view, could be related to neuroimaging abnormalities.

However, only few imaging studies investigated the anatomical correlates of cognitive insight in psychosis [43]. Specifically, Buchy and colleagues [47] found a direct association between cognitive insight and left hippocampal volume and an inverse correlation between C scores and bilateral hippocampal volumes. Moreover, R scores positively correlated with ventrolateral prefrontal cortex (VLPFC) gray matter volume in patients with psychosis [48] and with VLPFC activation in non-clinical subjects [39].

Therefore, the current study aimed to investigate the metabolic correlates of cognitive insight by using 18F-Fluoro-2-deoxyglucose Positron Emission Tomography (FDG-PET) scan and BCIS scores in patients with major psychosis, in order to further elucidate the anatomical bases of cognitive insight.

Although the lack of PET studies investigating the metabolic correlates associated with insight, several neuroimaging studies reported fronto-temporal alterations, especially in the DLPFC, VLPFC as well as in the inferior and superior temporal regions, as putative biomarkers of poor insight or weakness in cognition [49,50,51,52,53,54,55,56]. Therefore, we expected that our group of patients will show altered metabolism in the same fronto-temporal areas, based on the hypothesis that morphological deficits might also result in metabolic dysfunctions.

## 2. Methods and materials

### 2.1 Participants

Sixty-three inpatients (17 women and 46 men), aged between 18 and 65 years, with acute psychosis according to the DSM-IV-TR (APA, 2000) criteria were enrolled in the current study. Thirty-six were diagnosed with affective psychosis (all with bipolar disorder type I) and twenty-seven with non-affective psychosis (10 with schizophrenia, 17 with psychosis NOS) (for details see Tables 1 and 2).

**Table 1:** Characteristics of the affective psychosis sample (n = 36).

Diagnosis	Phase of illness	Gender (M/F)	Mean Age (SD^d^)	Mean Duration of Illness (SD^d^)	Chlorpromazine^e^ Mean Dosage (SD^d^)	Mean BPRS (SD^d^)	Mean MMSE (SD^d^)	Mean BCIS (SD^d^)
BD Type I n = 36	Depressive n = 3, Mixed n = 18, Manic n = 15	23/13	38,72 (12,22)	12,66 (10,55)	412,64 (388,44)	37,58 (5,71)	27,94 (1,86)	3,65 (5,72)

^d^ SD = standard deviation

^e^ Chlorpromazine dose (mg) equivalent of antipsychotics; BPRS: Brief Psychiatric Rating Scale; MMSE: Mini Mental State Examination; BCIS: Beck Cognitive Insight Scale.

**Table 2:** Characteristics of the non-affective psychosis sample (n = 27).

Diagnosis	First Episode (FE) vs chronic	Gender (M/F)	Mean Age (SD^d^)	Mean Duration of Illness (SD^d^)	Chlorpromazine^e^ Mean Dosage (SD^d^)	Mean BPRS (SD^d^)	Mean MMSE (SD^d^)	Mean BCIS (SD^d^)
SZ n = 10, NOS n = 17	FE n = 4, Chronic n = 23	23/4	32,77 (11,35)	12,33 (10,86)	939,95 (627,90)	38,10 (5,41)	27,62 (1,85)	3,44 (5,79)

^d^ SD = standard deviation

^e^ Chlorpromazine dose (mg) equivalent of antipsychotics; BPRS: Brief Psychiatric Rating Scale; MMSE: Mini Mental State Examination; BCIS: Beck Cognitive Insight Scale.

Exclusion criteria were cognitive deterioration (score lower than 24) as assessed by the Mini-Mental State Examination (MMSE) [57], consistent with normative data in the Italian population [58], or comorbidity with other neurological conditions involving Central Nervous System (e.g. cerebral tumors) and pregnancy or breastfeeding.

The study was approved by the local Ethical Committee (Comitato Etico, Fondazione “IRCCS Ca’ Granda” Policlinico di Milano) and informed written consent was obtained from all subjects. Capacity to consent was determined through clinical interview and MMSE, in order to assess the absence of cognitive deterioration.

## 2.2. Clinical assessment

Diagnoses were conducted at baseline with the Structured Clinical Interview for DSM-IV-TR, Axis I [59]. Socio-demographic data were collected by an interview completed by experts. Clinical assessment was carried out with the Brief Psychiatric Rating Scale (BPRS) to evaluate severity and change of psychotic and mood symptoms [60]. Cognitive insight was quantified with the BCIS self-report inventory [41]. The subscales “self-reflectiveness” (formed by 9 items) and “self-certainty” (formed by 6 items) are defined to measure separate aspects of cognitive insight. They rate the patients' ability to observe their mental productions and to reflect on alternative explanations and overconfidence in the validity of their beliefs. Accordingly, among psychotic patients an optimal cognitive insight is given by the results of high self-reflectiveness and low self-certainty; a composite index reflecting cognitive insight is the resulting difference between the two series. In the absence of cognitive insight, this index will have negative values [41,61].

### 2.3. FDG-PET scanning

FDG-PET scanning was performed within 7 days of insight and symptom assessment. Patients underwent FDG-PET at rest, after intravenous injection of 170 MBq. Each acquisition included a Computed Tomography (CT) transmission scan of the head (50mAs lasting 16 seconds) followed by a three-dimensional (3D) static emission of 15 minutes using a Biograph Truepoint 64 PET/CT scanner (Siemens). FDG-PET sections were reconstructed using an iterative algorithm (OS-EM), corrected for scatter and for attenuation using density coefficients derived from the low dose CT scan of the head obtained with the same scanner, with the proprietary software. Images were reconstructed in the form of transaxial images of 128×128 pixels of 2 mm, using an iterative algorithm, ordered-subset expectation maximization (OSEM). The resolution of the PET system was 4–5 mm FWHM.

### 2.4. Statistical analysis

The statistical analyses were performed using Statistical Parametric Mapping (SPM8, Wellcome Department of Cognitive Neurology, London, UK). FDG PET data were subjected to affine and nonlinear spatial normalization into the MNI space. The spatially normalized set of images was smoothed with an 8mm isotropic Gaussian filter to blur for individual variations in gyral anatomy and increase the signal-to-noise ratio. Images were globally normalized to 50 using proportional scaling to remove confounding effects to global cerebral glucose consumption changes, with a masking threshold of 0.8.

Subjects were divided into four groups considering the BCIS score (low insight with R-C = <5 and high insight with R-C>5, see Table 3) and the diagnosis (affective vs non affective psychosis). The choice of the cut-off of 5 to divide the two FDG-PET groups with different BCIS composite index score (= <5 and >5) was motivated by the average value (4,64) shown in psychotic patients in the literature [61], similar to the median BCIS scores of our group of patients. We performed two-sample t-test between the low and the high insight groups to determine if the data were significantly different, considering P values < 0.05 as statistically significant. We found no statistical differences between the two groups in any of the demographic or clinical variables employed in this study, including age (p = 0,335), sex (χ^2^ = 0,544, p = 0,461) duration of illness (p = 0,123) and the BPRS scores (p = 0,142). Finally, we found no significant differences between the two groups for the diagnosis (χ^2^ = 0,049, p = 0,825).

**Table 3:** Characteristic of the high and low insight samples.

	Diagnosis	Gender (M/F)	Mean Age (SD^d^)	Mean Duration of Illness (SD^d^)	Mean BPRS (SD^d^)	Mean MMSE (SD^d^)
**High insight n = 27**	12 SZ, 15 BD	6 F, 21 M	37.89 (12.16)	14.85 (12.52)	36.4 (5.53)	28.32 (1.75)
**Low insight n = 36**	15 SZ, 21 BD	11 F, 25 M	34.89 (12.12)	10.69 (8.58)	38.58 (5.73)	27.38 (1.84)
**P value**	p = 0.825	p = 0.461	p = 0.335	p = 0.123	p = 0.142	p = 0.044

^d^ SD = standard deviation; BPRS: Brief Psychiatric Rating Scale; MMSE: Mini Mental State Examination.

Significant differences in brain metabolism among the four groups (non affective psychosis with low insight, non affective psychosis with high insight, affective psychosis with low insight, and affective psychosis with high insight) were estimated by means of single-subject, conditions and covariates design, with the 4 groups of psychosis modeled as conditions, with insight score as covariate variable of all patients and age as the nuisance variable for group analyses. First, we performed a one-way analysis of variance (ANOVA) between the four groups. Then, we performed two-sample t-test between pair of groups with low insight compared to those with high insight.

All voxel-based statistical analyses of brain glucose metabolism images were performed by applying a statistical threshold of uncorrected p<0.005 at voxel level for F-test and p<0.005 corrected at cluster level for t-test, with an extent threshold of at least 35 contiguous voxels. P values < 0.005 were considered statistically significant in analysis of covariance to correlate the cognitive insight score with the metabolism.

## 3. Results

The comparison between the four groups (non affective psychosis with low and high insight and affective psychosis with low and high insight), showed significant metabolic differences in the fusiform gyrus, superior temporal gyrus and insula, in the right hemisphere, and in the precuneus and superior temporal gyrus and insula, in the left hemisphere (ANOVA, p<0.005). Several post-hoc analyses were carried out to investigate the direction of these abnormalities across the four different groups (see Table 4).

**Table 4:** Anatomic locations, side, spatial extent of the clusters in voxels, Montréal Neurological Institute (MNI)-space coordinates of the statistical peaks, F scores (ANOVA, upper panel) or T scores (two-sample t tests, middle panel) and *Z* scores of the brain areas showing significant differences of metabolism (p<0.005) in the entire group (or in the non-affective subgroup or in the affective subgroup) between the one with low insight and the one with high insight, and correlation (lower panel) between the cognitive insight score and metabolism (p<0.005). L, left; R, right.

***One-way ANOVA***	**Side**	**Cluster size**	***F* value**	***Z* score**	**MNI**^**d**^ **coordinates *(x*, *y*, *z)***		
**Fusiform gyrus**	R	56	14.34	3.37	34	-50	-16
**Superior temporal gyrus**	L	77	13.66	3.29	-60	-16	12
**Insula**		subcluster	11.38	3.00	-38	-6	8
**Superior temporal gyrus**	R	49	12.38	3.14	56	-10	8
**Precuneus**	L	35	11.40	3.01	-12	-44	46
**Insula**	R	35	11.38	3.00	42	-6	-4
***Post-hoc two-samples T-test Low Insight vs*. *High Insight***	**Side**	**Cluster size**	***T* value**	***Z* score**	**MNI**^**d**^ **coordinates *(x*, *y*, *z)***		
***Hypermetabolism in entire group***							
**Orbito-frontal gyrus**	L	38	3.48	3.30	-12	38	-24
***Hypometabolism in entire group***							
**Fusiform gyrus**	R	70	3.79	3.56	34	-50	-16
**Superior temporal gyrus**	L	139	3.70	3.48	-60	-16	12
**Insula**	L	subcluster	3.37	3.21	-38	-6	8
**Superior temporal gyrus**	R	70	3.52	3.33	56	-10	8
**Precuneus**	L	41	3.22	3.07	-12	-44	46
**Insula**	R	84	3.11	3.02	44	-6	-4
***Hypermetabolism in non affective***							
**Orbito-frontal gyrus**	L	63	3.51	3.33	-12	38	-25
***Hypometabolism in non affective***							
**Precuneus**	L	138	4.05	3.78	-12	-46	48
**Superior temporal gyrus**	L	174	3.85	3.61	-60	-16	12
**Insula**	L	subcluster	3.30	3.14	-38	-6	6
**Superior temporal gyrus**	R	94	3.81	3.58	56	-10	8
**Insula**	R	111	3.18	3.04	44	-4	-4
***Hypermetabolism in affective***							
**Orbito-frontal gyrus**	R	39	3.29	3.13	44	36	-6
***Hypometabolism in affective***							
**Inferior temporal gyrus**	R	152	4.01	3.75	56	-44	-16
***Correlation with Insight-score***	**Side**	**Cluster size**	***T* value**	***Z* score**	**MNI**^**d**^ **coordinates *(x*, *y*, *z)***		
**Precuneus**	R	52	3.40	3.23	8	-58	34

^d^ MNI = Montréal Neurological Institute.

**Fig 1:**
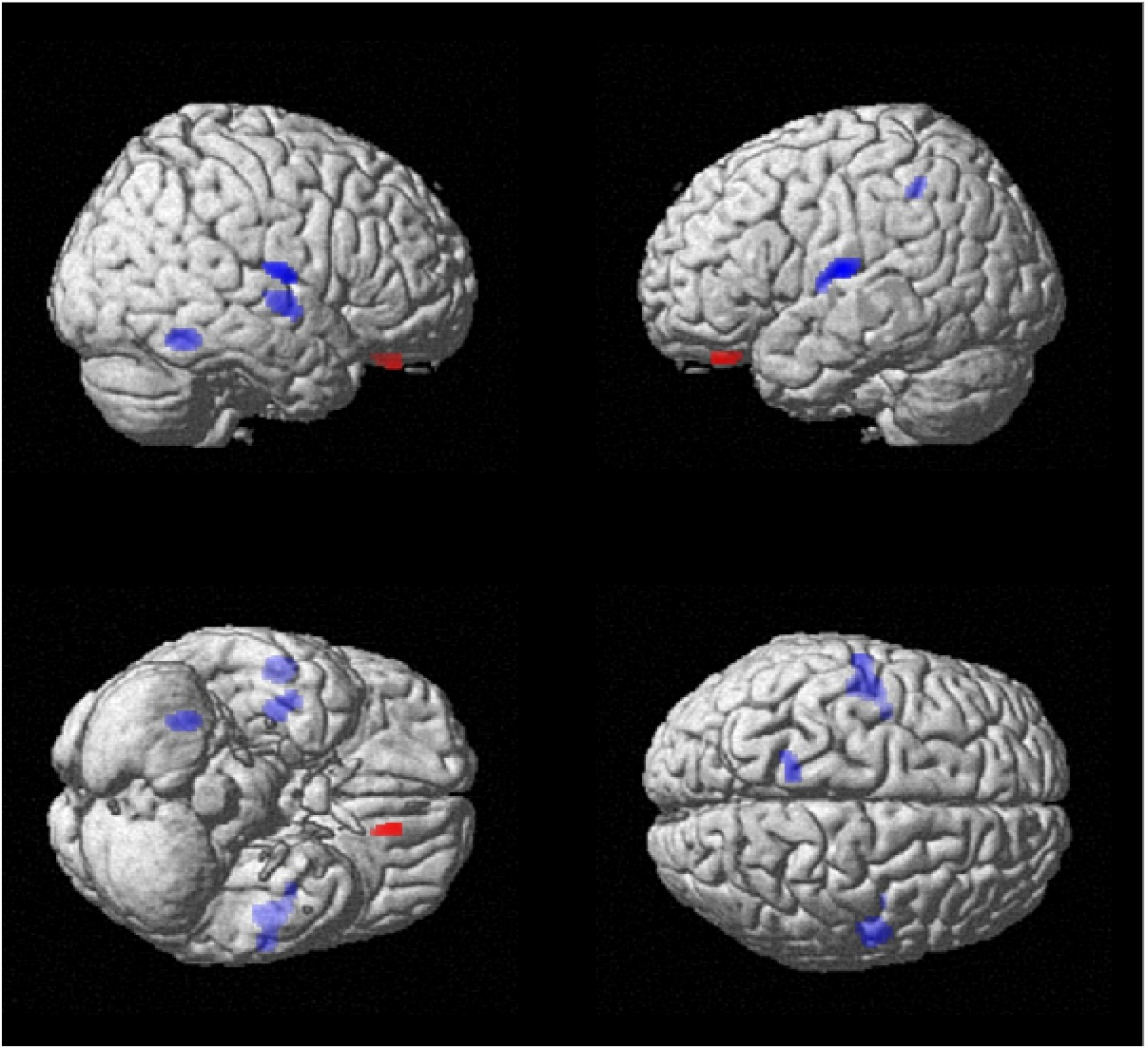
Statistical map of the post-hoc two-sample t-test among the groups of psychosis with low insight versus psychosis with high insight, overlaid upon the average MRI in stereotaxic space, showing decreased metabolism (in blue color) in right fusiform gyrus, superior temporal gyrus and insula and in left precuneus and superior temporal gyrus and insula and increased metabolism (in red color) in left orbito-frontal gyrus (all p<0.005).

The post-hoc two-samples t-test analyses, between the entire group with low insight compared to the one with high insight, revealed in the group with low insight decreased metabolism (p<0.005) in fusiform gyrus, superior temporal gyrus and insula, in the right hemisphere, and in precuneus and superior temporal gyrus and insula, in the left hemisphere, and showed increased metabolism in left orbito-frontal gyrus (p<0.005) (see Fig 1).

The post-hoc two-samples t-test analyses, between the non-affective group with low insight compared to the one with high insight, revealed that the group with low insight showed decreased metabolism in right superior temporal gyrus and insula, in left precuneus, in left superior temporal gyrus and insula as well as increased metabolism in left orbito-frontal gyrus (all p<0.005).

The post-hoc two-samples t-test analyses, between the affective group with low insight, compared to the one with high insight, revealed in the group with low insight decreased metabolism in the right inferior temporal gyrus and increased metabolism in right orbito-frontal gyrus (all p<0.005).

Finally, the analysis of covariance showed a positive significant correlation (p<0.005) between the metabolism in right precuneus and insight scores.

## 4. Discussion

Our discussion focuses first on areas in which we observed hypometabolism and a correlation between insight score and low metabolism. Our results suggest that a reduced posterior (occipito-temporo-insulo-parietal) and increased anterior (orbito-frontal) cerebral metabolism sustains low cognitive insight in psychosis. The analyses were carried out comparing four groups, non affective psychosis with low and high insight and affective psychosis with low and high insight. Moreover we decided to perform several post-hoc analyses to investigate the direction of these abnormalities across the four different groups. All the patients had psychotic symptoms and were in acute phase at recruitment. Additionally, the direct comparison between BD and SZ did not show statistical significant differences on cognitive insight (R-C) scores. Therefore, based on these evidence, patients were considered as one homogeneous sample split into high and low cognitive insight. Indeed, it has been consistently reported that psychosis is a trans-diagnostic dimension between in SZ and BD [62]. Both disorders share extensive similarities in terms of biological, clinical, genetic and neurocognitive factors, suggesting a continuous distribution of symptoms among the two disorders [62,63,64,65,66,67,68].

With regards to our findings, patients reported decreased cerebral metabolism in extensive regions encompassing occipital (fusiform gyrus), parietal (precuneus), and temporal (superior temporal gyrus and insula) areas. Specifically for occipital regions, the fusiform gyrus is thought to be involved in face recognition and seems to be associated with prosopagnosia (impaired facial recognition) [69]. Interestingly, previous studies have also reported that face recognition might be related to insight by showing an association between this capacity and self-awareness which may, therefore, account for the known difficulties of SZ patients in elaborating a coherent self experience [70,71].

Hypometabolism was also found in the superior temporal gyrus bilaterally. The superior temporal cortex has been studied for its main role in receptive language [72,73], social cognition, in the Theory of Mind and processing of social stimuli [74,75,76]. Additionally, Buchy and colleagues [39] hypothesized that cognitive insight is based on accurate memory for specific life experiences which in turn affects everyday beliefs and judgments, suggesting therefore a possible role for temporal areas.

Moreover neuroimaging studies found structural and functional abnormalities in the superior temporal gyrus among SZ patients [77,78,79] and decreased gray matter volume in prefrontal and temporal regions which related to low insight scores in a group of first-episode drug naïve psychotic patients, in comparison with a control group [80]. Nevertheless, to date, the role of this area in insight is still not well elucidated.

In contrast, lack of insight in patients with non affective psychosis has been frequently related to impairment in the prefrontal and parietal cortices [34,81]. Therefore, decreased metabolism, together with the positive correlation between metabolism and insight scores in the precuneus, and increased metabolism in the orbital frontal cortex (OFC) seem to confirm this evidence. Furthermore, these findings are not surprising given that the precuneus, a part of the superior parietal lobule, plays a role in a wide range of integrated tasks, including visuo-spatial imagery, which is particularly related to representation of other's perspective, episodic and autobiographical memory retrieval and self-processing [49,82]. Additionally, this area seems to be also implicated in self-consciousness, through a hypothetical neurobiological pathway of impaired clinical and cognitive insight in SZ, as recently described by Xavier and Vorderstrasse [83].

Indeed, it has been shown that both perfusion and activation of bilateral precuneus correlated to insight both in SZ and in BD patients [84,85].

Therefore, based on this evidence, altered precuneus activation could indirectly reflect a reduced integration between past and current information related to self and other reflective processes and to cognitive insight [85]. It could also support an altered self experience, which could correlate with deficits in metacognitive abilities, shown to be associated to insight [32,33,34,35,36].

Similarly, the OFC plays a major role in many neuropsychological processes [86] and it might therefore be also associated with insight. Specifically OFC is described in literature as a polymodal brain region that receives inputs from many different sensory modalities [87] and brain alterations in this area have been linked to several psychiatric disorders [88], including SZ and BD [86,89,90,91,92,93]. Moreover, OFC is considered, with its adjacent areas, a neural substrate of social behavior, critical for decision making and probably for intuitive processes [94,95]. Indeed it has been shown that damage in this area causes cognitive, affective and social impairments [96].

Even though most studies have shown cognitive insight to be related to frontal lobe effectiveness [97,98] the current study highlights that more brain areas, rather than the only prefrontal cortex, are involved in cognitive insight process. Other brain imaging studies have shown the interaction between frontal and posterior cortex in metacognition [99]. Fleming & Dolan [100] suggested a neural combination in which prefrontal regions interact with cingulate and insula to promote accurate judgments of performance; Moran et al. [101] described a self-reflective process network that includes dorsomedial and ventromedial prefrontal cortex, anterior and posterior cingulate, anterior insula, inferior frontal gyrus, temporo-parietal junction/AG and inferior parietal lobule. A latest study in healthy subjects identified a hypothetical cognitive insight’s functional neural basis in the ventrolateral prefrontal cortex, in temporal and occipital cortices [39].

We also assume that self-reflection and reduced self-certainty should be a focal point for poor cognitive insight in psychosis, representing a complex phenotype potentially sustained by a combination of neurobiological vulnerability (genes, neural circuitry formed by prefrontal, cingulate, temporal and parietal cortices, insula, hippocampus) and environment [83]. Understanding cognitive distortions and neural correlates might be of paramount importance in psychosis because they could be a predictor of good prognosis.

The results of our study should be interpreted cautiously due to some limitations. First, our sample was 73% male and did not consent to discover sex effects in cerebral metabolism–cognitive insight associations. Moreover, the large age range could have led to a potential confounding effect, therefore we decided to use this parameter as a covariate in the statistical analysis. Second, a healthy control group was lacking limiting the generalizability of our results. Third, neuropsychological measures were not collected. Therefore future studies should take into account specific assessment of executive functions to identify their impact on cognitive insight. Finally, since BD is characterized by swinging mood episodes, a cross sectional assessment of metabolic correlates of cognitive insight may not be fully generalizable.

In conclusion, our findings suggest reduced posterior (occipito-temporo-parietal cortices and insula) and increased anterior (orbito-frontal) cerebral metabolism supporting low cognitive insight in psychosis, especially in the right emisphere.

This is the first study that takes into account the cognitive insight and brain metabolism of affective and non-affective psychotic patients. Future longitudinal studies should explore cognitive insight over time in psychosis before and after treatment, coupling with comprehensive cognitive evaluation.

## Supporting information

S1 Dataset Dataset. (XLS)

S1 Fig Insight PET3D-ttest-affective. (TIF)

S2 Fig Insight PET3D-anova-ftest. (TIF)

S3 Fig Insight PET3D-ttest-nonaffective. (TIF)

S4 Fig Correlation insight. (TIF)
