# Supplementary figures and images for: The metabolic basis of cognitive insight in psychosis: A positron emission tomography study
Source: PLoS One. 2017 Apr 17;12(4):e0175803. doi: 10.1371/journal.pone.0175803 (PMC5393874; doi:10.1371/journal.pone.0175803)

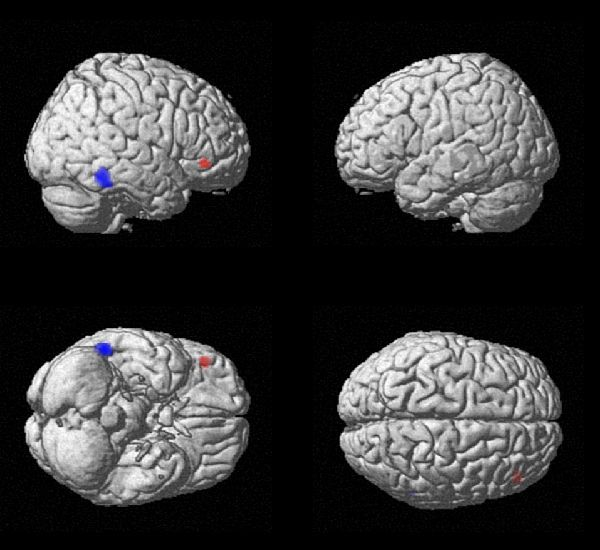

Supplement: S1 Fig — (TIF) [file pone.0175803.s002.tif]

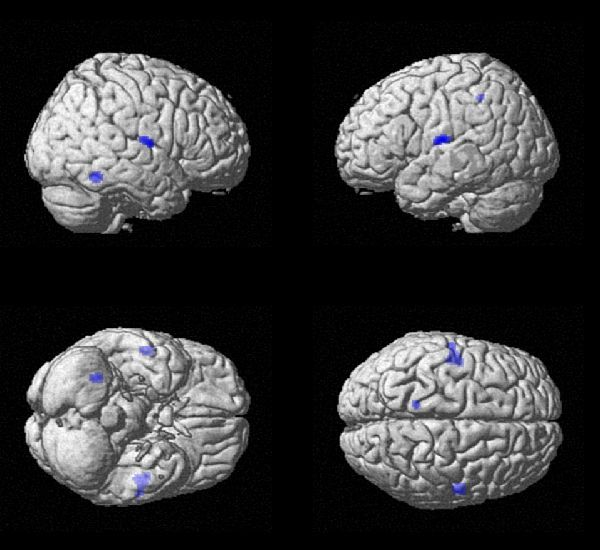

Supplement: S2 Fig — (TIF) [file pone.0175803.s003.tif]

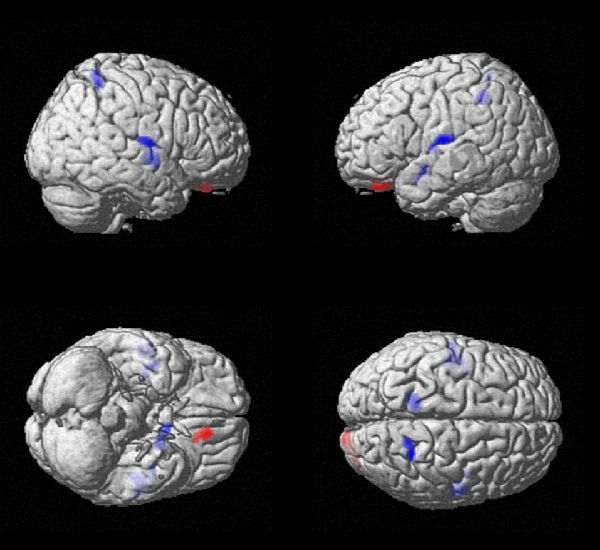

Supplement: S3 Fig — (TIF) [file pone.0175803.s004.tif]

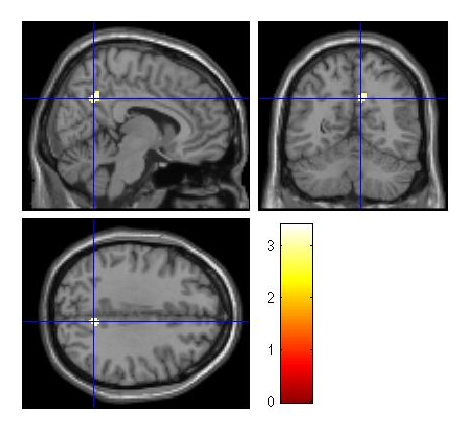

Supplement: S4 Fig — (TIF) [file pone.0175803.s005.tif]
